# Extracellular Juxtamembrane Motif Critical for TrkB Preformed Dimer and Activation

**DOI:** 10.3390/cells8080932

**Published:** 2019-08-19

**Authors:** Jianying Shen, Dang Sun, Jingyu Shao, Yanbo Chen, Keliang Pang, Wei Guo, Bai Lu

**Affiliations:** 1 School of Pharmaceutical Sciences, IDG/McGovern Institute for Brain Research, Tsinghua University, Beijing 100084, China; 2 Artemisinin Research Center, Institute of Chinese Materia Medica, China Academy of Chinese Medical Sciences, Beijing 100084, China; 3 R & D Center for the Diagnosis and Treatment of Major Brain Diseases, Research Institute of Tsinghua University in Shenzhen, Shenzhen 518057, China

**Keywords:** receptor tyrosine kinases, TrkB, preformed dimer, extracellular juxtamembrane motif, antibodies, brain-derived neurotrophic factor

## Abstract

Receptor tyrosine kinases are believed to be activated through ligand-induced dimerization. We now demonstrate that in cultured neurons, a substantial amount of endogenous TrkB, the receptor for brain-derived neurotrophic factor (BDNF), exists as an inactive preformed dimer, and the application of BDNF activates the pre-existing dimer. Deletion of the extracellular juxtamembrane motif (EJM) of TrkB increased the amount of preformed dimer, suggesting an inhibitory role of EJM on dimer formation. Further, binding of an agonistic antibody (MM12) specific to human TrkB-EJM activated the full-length TrkB and unexpectedly also truncated TrkB lacking ECD (TrkBdelECD365), suggesting that TrkB is activated by attenuating the inhibitory effect of EJM through MM12 binding-induced conformational changes. Finally, in cells co-expressing rat and human TrkB, MM12 could only activate TrkB human-human dimer but not TrkB human-rat TrkB dimer, indicating that MM12 binding to two TrkB monomers is required for activation. Our results support a model that TrkB preforms as an inactive dimer and BDNF induces TrkB conformation changes leading to its activation.

## 1. Introduction

Receptor tyrosine kinases (RTKs), such as the receptors for EGF, FGF, PDGF, etc., are all single transmembrane receptor proteins. They relay the signals of extracellular factors to intracellular signal transduction mechanisms [1,2,3,4]. For RTK activation, a dogmatic view is the ligand-induced dimerization model, in which binding of a dimeric ligand to the receptor protein would induce the dimerization of the receptors, leading to the activation of its intracellular tyrosine kinase domain and intra-molecular tyrosine phosphorylation [5,6]. This would trigger a cascade of signaling events, leading to many cellular and tissue processes [1,2,3,4]. However, there is evidence suggesting that ErbB, the receptor for EGF, may exist as inactive dimers on the cell surface before ligand binding [7,8,9,10,11,12,13,14,15,16], challenging the dogmatic view. Crystal structure data on the extracellular domain (ECD) of ErbB receptor have shown that ligand-binding to the ECD of the receptor dimer induced its conformational changes [17,18,19,20,21,22], leading to the activation of this RTK. It is not widely known whether other receptor tyrosine kinases could also exist as preformed dimers.

Trk receptors (TrkA, TrkB and TrkC), the receptors for the neurotrophins NGF, brain-derived neurotrophic factor (BDNF)/NT-4, NT-3 (TrkA and TrkB also bind NT-3, but to a lesser extent), respectively, belong to another family of RTKs [23,24,25]. Similar to other RTKs, Trk consists of an extracellular domain, a single transmembrane region and an intracellular kinase domain [26]. The organization of the extracellular domain from N-terminus to C-terminus is: a leucine-rich region flanked by two cysteine-rich regions named D1 to D3, two immunoglobulin-like domains named D4 and D5. A spacer linking D5 with the transmembrane domain is termed as an extracellular juxtamembrane motif (EJM) [27,28]. Upon binding to its ligands, Trk receptors activate various intracellular signaling cascades including Akt, Erk1/2 and PLCγ [5,29].

Despite extensive studies, the molecular mechanism underlying Trk receptor activation remains elusive. Early work suggests that NGF binding induced TrkA receptor dimerization and phosphorylation [26,30,31], and the NGF homodimer is thought to serve as a bridge to bring the two TrkA monomers together [32]. This ligand-induced dimer model is supported by the crystallographic study showing that the two D5 regions could be co-crystalized with an NGF dimer [33]. However, using chemical crosslinking and protein fragment complementation assays, we have previously shown that TrkA and TrkB could exist as inactive and preformed dimers in living cells in heterologous expression systems [34,35].This preformed dimer model was consistent with the recent crystal structures of inactive, symmetric kinase domain dimers of TrkA and TrkC [27,36].

In the present study, we demonstrated that in the cultured primary neurons, endogenous TrkB exists primarily as inactive and preformed dimers, and identified key extracellular domains critical for the interaction of TrkB preformed dimers. Further, we found that the EJM is an inhibitory motif for TrkB dimer formation, and showed that molecular perturbation on the EJM results in conformational changes, leading to TrkB activation. Our findings suggest the classical ligand-induced dimerization model should be re-evaluated.

## 2. Materials and methods

### 2.1. Materials

BDNF was obtained from Sino Biological (Beijing, China). MM12 antibody was a gift from Sino Biological (Beijing, China). Antibodies against TrkB, phosphorylated TrkB (Tyrosine in 515/706/707/816) were purchased from Cell Signaling Technology (Danvers, MA, USA). Antibody against GAPDH was obtained from Easybio. HRP-conjugated goat anti-rabbit IgG and anti-mouse IgG were from Thermo Scientific (Rockford, IL, USA). The BCA protein assay kit and the Pierce ECL western blotting substrate were obtained from Thermo Scientific. CHO and CHO with stable transfected human TrkB (CHO-hTrkB) cell lines were purchased from Life Technologies (Foster, CA, USA). PC12 cell lines were purchased from Peking Union Medical College. PC12-rTrkB cell line was a gift from Moses Chao. All cell culture media and reagents were obtained from Gibco.

### 2.2. Construction of Expression Vectors

pGFP-hTrkB was a gift from Maruyama lab in Okinawa Institute of Science and Technology [8] and was used as a template. eGFP was amplified using a primer pair: BglII-EcoRI-XbaI-(G4S)3-GFP-F (GCAGATCTGAATTCTCTAGAGGTGGTGGTTCTGGTGGTGGTT CTGGTGGTGGTTCTATGGTGAGCAAGGGCGAGGAGCTGTTCAC) and XhoI-stop-GFP-R (GCCTCGAGCTACTACTTGTACAGCTCGTCCATGCCGAGAGTG) and the PCR product was inserted into pGFP-hTrkB between BglII and XhoI. Then hTrkB was amplified using primer pair: EcoR1-hTrkBFL-F (TCTGAATTCTGTCCCACGTCCTGCAAATGCAGTGCCTCTCGGATCTG) and XbaI-hTrkB-R (ACCTCTAGAGCCTAGAATGTCCAGG TAGACCGGAGATGC) and the PCR product was inserted between EcoRI and XbaI. The hTrkBdelECD365-eGFP was amplified using a primer pair: TCTGAATTCGAGTATGGGAAGGATGAGAAACAG and ACCTCTAGAGCCTA GAATGTCCAGGTAGACCGGAGATGC, and the PCR product was inserted between EcoRI and XbaI. rTrkB-eGFP plasmid was constructed by inserting PCR product of rTrkB between EcoRI and XbaI. rTrkBhEJM plasmid was constructed by exchanging rTrkBEJM (GPPGVDYETNPNYPE VLYEDWTTPTDIGDTTNKSNEIPSTDVADQTNREH) into hTrkBEJM (GWPGIDDGANPNYPD VIYEDYGTAANDIGDTTNRSNEIPSTDVTDKTGREH) using Hieff MutTM Site-Directed Muragenesis Kit (Yeason, Shanghai, China).

### 2.3. Cell Culture

Culture of hippocampal neurons from embryonic (E18 days) rat embryos was performed. Neurons were dissociated and plated on 100 ng/mL poly-D-lysine-coated 12-well plates at 300, 000 cells/well. Neurons were cultured for 10–12 days, then treated by reagent and processed for western blotting. CHO cells were cultured in F12k media supplemented with 10% fetal bovine serum, 50 units/mL penicillin G, 50 μg/mL streptomycin. PC12 cells were cultured in DMEM media with 5% fetal bovine serum and 10% horse serum. All cells were maintained at 37 °C in a humidified atmosphere of 95% air and 5% CO_2_.

### 2.4. Primary Neuron Culture

Animal experiment was carried out in accordance with the recommendations of Association for Assessment and Accreditation of Laboratory Animal Care International (AAALAC). The pregnant rats were euthanized following IACUC protocol. Rat hippocampal neurons (embryonic day 18) were dissociated with 1 mL 0.25% trypsin (1:1, Life Tech) in Hank’s Balanced Salt Solution (HBSS, Life Tech) at 37 °C. After 30 min incubation, the enzyme solution was removed and washed in warmed DMEM, with 10% FBS added to stop the enzymatic digestion. Cells were then plated on 18-mm poly-D-lysine–coated coverslip at 250,000 cells per well in 24-well plates. After overnight incubation, the culture medium was replaced with NeuroBasal medium (Invitrogen, Waltham, MA, USA) with 2% B-27 (Invitrogen) and 1% GlutaMAX-I (Invitrogen) [37]. Neurons were used at 10-12 DIV.

### 2.5. Transient and Stable Transfection

hTrkB-eGFP, rTrkB-eGFP, hTrkBdelECD365-eGFP, or rTrkBhEJM was transfected to CHO cells or PC12 cells using lipofectamine 2000 reagent (Life technologies) according to the manufacturer’s protocol. Briefly, 1 × 10^5^ cells in 1 mL medium in 12-well plate were cultured for 24 h before transfection. Then mix about 12 μg DNA with 150 μL opti-MEM medium and mix bout 12 μL of lipofectamine 2000 reagent with 150 μL opti-MEM medium respectively. Next, diluted DNA was added to diluted transfection reagent by 1:1 ratio and incubated with 5 min in the room temperature. In the end, the DNA-lipid complex was added to cells. Replace the culture medium with new medium added with 1.0 mg/mL of G418 after about 48 h. The stable expressing cell lines were obtained under the selection of G418 after about 3 weeks.

### 2.6. Native PAGE and Western Blotting

Cells were lysed in the lysis buffer (20 mM HEPES pH7.4, 150 mM NaCl, 1% NP-40, 2 mM EDTA and a phosphotase inhibitor cocktail purchased from Roche (Basel, Switzerland). Two micro grams of total protein was mixed with the loading buffer without SDS and β-ME. The separating gel and stacking gel were made without SDS. The running buffer was in pH 8.3 without SDS. The gel was powered up at a current of 20 to 25 mA constant current. The gel was run until the bromophenol blue dye front reached the bottom. Then, samples were transferred to PVDF membrane for 120 min at 300 mA. After transfer, the membrane was incubated in 20 mL of 8% acetic acid for 15 min to fix the protein. The membrane was blocked with 5% bovine serum albumin (BSA) for 1 h at room temperature. Then, the membrane incubated with the primary antibodies in blocking buffer at 4 °C overnight before detection with HRP-conjugated secondary antibodies. Chemiluminescence was detected with ECL solution. Every test was repeated at least twice.

### 2.7. SDS-PAGE and Western Blotting

Cells were lysed in the lysis buffer (20 mM HEPES pH7.4, 150 mM NaCl, 1% NP-40, 1% sodium deoxycholate, 0.1% SDS, 2 mM EDTA and a phosphoprotease inhibitor cocktail purchased from Roche). Samples were mixed with the loading buffer, incubated at 100 °C for 5 min and resolved by SDS-PAGE. Then samples were transferred to PVDF membrane for 120 min at 300 mA. The membrane was blocked with 5% BSA for 1 h at room temperature. Then the membrane incubated with the primary antibodies in blocking buffer at 4 °C overnight before detection with HRP-conjugated secondary antibodies. Chemiluminescence was detected with ECL solution. Every test was repeated at least twice.

### 2.8. Protein Expression and Purification

hTrkB gene copy was bought from Addgene as pDONR223-NTRK2 (Plasmid #23883). Then TrkB ECD, amino acid 32~430 (whole ECD) and 32~365 (Domain 1 to Domain 5), were subcloned into pFastBac Dual after the polyhedrin promoter. A gp67 signal peptide was introduced at the N-terminal of TrkB ECD for protein secretion and 6-histidine tag was introduced at the C-terminal for affinity purification. The Bac-to-Bac expression system from Invitrogen was used to make baculovirus containing TrkB ECD gene.

Baculovirus-infected SF9 cells were cultured in MSF1, from Sino Biological (Beijing, China) at 27 °C. Supernatant was harvested 72 h after infection by centrifugation at 6000× *g* for 10 min. Affinity purification was performed by Ni-NTA column (HisTrap excel, GE Healthcare). The column loaded with the supernatant was washed with a buffer containing 150 mM NaCl, 25 mM Tris-HCl, pH 8.0. TrkB ECD was eluted by a buffer containing 500 mM imidazole, 150 mM NaCl, 25 mM Tris-HCl, pH 8.0. The eluent was collected, concentrated and further purified by size-exclusion chromatography (Superdex-200, GE Healthcare) in 150 mM NaCl, 25 mM Tris-HCl, pH 8.0. Peak fractions containing TrkB ECD were collected and saved at −80 °C for further experiments.

## 3. Results

### 3.1. Endogenous TrkB Is Expressed Largely as Preformed Dimers in Cultured Primary Neurons

**Figure 1:**
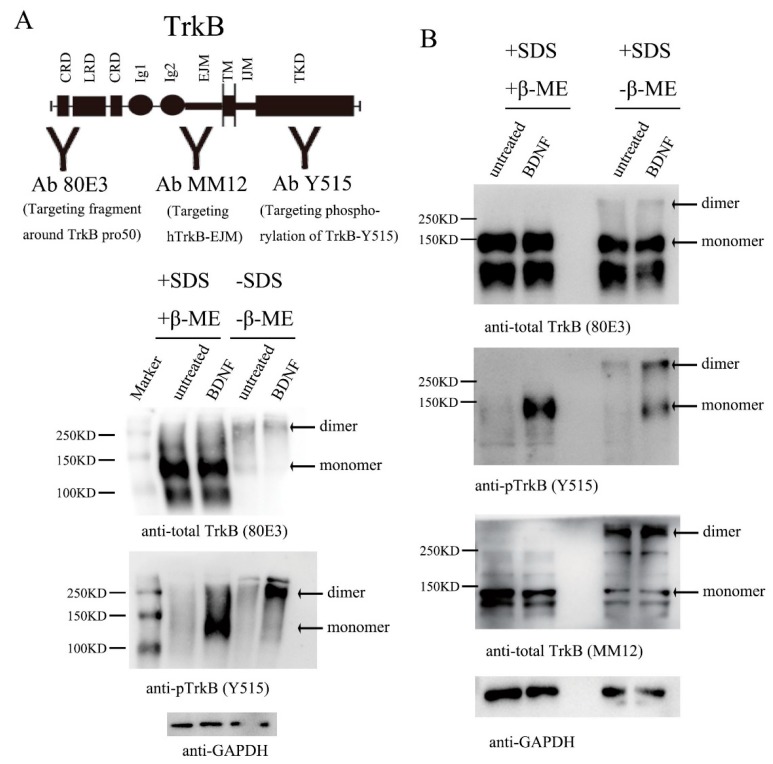
TrkB exists as an inactive and preformed dimer that is activated by brain-derived neurotrophic factor (BDNF) in primarily culture neurons. Rat hippocampus primary culture neurons maintained at 37 °C for 12 days and stimulated with vehicle or BDNF (1.0 nM) for 15 min. The lysates were treated in the presence (+) or absence (−) of SDS, with (+) or without (−) β-ME. Equal amounts of lysate proteins (~2 μg) were loaded in all lanes, and Western blots were performed after gel electrophoresis, using antibodies as indicated. Similar experiments were performed at least three times, and representative blots are presented. (**A**) Top: a diagram of whole TrkB, showing different domains as well as the sites where different antibodies bind. CRD: cysteine-rich domains; LRD: leucine-rich domain; Ig1 and Ig2: immunoglobulin-like domain 1 and 2; EJM: extracellular juxtamembrane domain; TM: transmembrane domain; IJM: intracellular juxtamembrane domain; TKD: tyrosine kinase domain. GAPDH: Glyceraldehyde 3-phosphate dehydrogenase. Middle: Existence of TrkB dimer in cultured primary neurons. Note that in native gel without β-ME (the right two lanes), TrkB existed primarily as a dimer. Bottom: activation of TrkB dimer. The same experiment as above was performed, but the blot was probed with an anti-pTrkB antibody, showing activated TrkB. (**B**) Role of disulfide bond in TrkB preformed dimer. All experiments were performed the same way as in A, except lysates were all denatured by SDS, with or without β-ME. Western blots were probed with antibodies 80E3 (top), anti-pTrkB (Y515) (middle), and MM12 (bottom). Note that disruption of disulfide bond by β-ME completely eliminated TrkB dimer.

Previous studies have shown that TrkA or TrkB could exist as inactive, preformed dimers in heterologous cells transfected with TrkA or TrkB [34,35]. Do preformed dimers also exist endogenously? In this study, we first investigated whether endogenous TrkB preformed dimer exists in the absence of its ligand BDNF in cultured primary neurons. The rat embryo hippocampal neurons (E18) were dissociated and cultured for 12 days and treated with or without BDNF (1.0 nM). Cell lysates were harvested without any denaturing agents and subjected to native polyacrylamide gel electrophoresis (PAGE), in the absence of the denaturing agent sodium dodecyl sulfate (SDS) and reducing agent β-mercaptoethanol (β-ME). A number of antibodies against TrkB or phosphorylated TrkB (pTrkB) were used in these biochemical experiments (Figure 1A, top). Using an antibody that detected the N-terminal portion of TrkB (80E3), we observed two bands: a 140 kDa band and a 280 kDa band, corresponding to TrkB monomer and dimer, respectively (Figure 1A, middle-right, and Supplementary Figure S1B). Western blots using an antibody against TrkB EJM (MM12) produced almost identical results (Supplementary Figure S1A). Thus, in the native gel, almost all TrkB molecules existed as dimers. However, the TrkB dimers were largely disrupted by SDS and β-ME (Figure 1A, middle-left, Figure 1B-left, top and bottom panels). Further, application of BDNF dramatically increased the dimer (native gel) and monomer (in SDS and β-ME) of phosphorylated TrkB (Figure 1A, bottom, Supplementary Figure S1C,D). However, treatment with BDNF did not change the amount of TrkB dimer (Figure 1A, middle, Figure 1B, middle, Supplementary Figure S1A,B). The goal of this and subsequent experiments was to obtain qualitative observation, rather than quantitative measurements. We therefore did not perform the experiments exactly the same way for quantitative densitometry. Instead, we performed similar experiments multiple times using different TrkB antibodies, and similar results were obtained. Interestingly, antibody 80E3 detected much less TrkB monomer in the absence of SDS (compare Figure 1A middle-left panel and Figure 1B top-right panel, also compare Supplementary Figure S1B,D bottom). Since the only difference between the two panels is whether it included SDS or not, it is possible that the antibody binds TrkB monomer much better in its denaturing form (in SDS). Nevertheless, it is important to note that treatment of neurons with BDNF resulted in a marked increase in phosphorylated TrkB dimer as seen in the native gel, suggesting the preformed inactive dimer on neuronal membraned could be activated by ligand stimulation.

Next we examined whether the disulfide bond was necessary for the maintenance of the TrkB dimer. The cell lysate was resolved on SDS-PAGE in the presence or absence of β-ME. Again, the TrkB monomer and dimer were observed as 140 kDa and 280 kDa bands, respectively, when the cell lysate was blotted with anti-TrkB antibodies, 80E3 and MM12 (Figure 1B, top and bottom). Phosphorylated TrkB monomer and dimer were detectable only in the cells treated with BDNF (Figure 1B, middle). The disulfide bond was found to be necessary for dimer maintenance, as the TrkB dimer was disrupted by β-ME treatment (Figure 1B, compare left two lanes with right two lanes). In the absence of β-ME, not all TrkB existed as dimers. Interestingly, the ratio of TrkB dimers to monomers were different when detected by two different monoclonal antibodies (Figure 1B, top and bottom). Antibody MM12, which recognizes TrkB EJM (Figure 1A, top), could react more TrkB dimers than monomers (Figure 1B bottom). In contrast, antibody 80E3, which recognizes region surrounding Pro50 of TrkB (Figure 1A, top), detected primarily monomers (Figure 1B, top). These results suggest that either 80E3 has a reduced affinity for TrkB dimer or MM12 has a reduced affinity for TrkB monomer under the reducing conditions (+β-ME). It was noted that under the denaturing conditions (+SDS) 80E3 also detected a 95 kDa band (Figure 1A middle and Figure 1B top), a position similar to that of truncated TrkB, TrkB-T1 expressed in cultured primary neurons. Given that this band was not seen by MM12, which in theory should also bind TrkB-T1, the nature of the 95kDa band remains to be determined.

### 3.2. TrkB EJM Inhibits the Formation of Preformed Dimers

**Figure 2:**
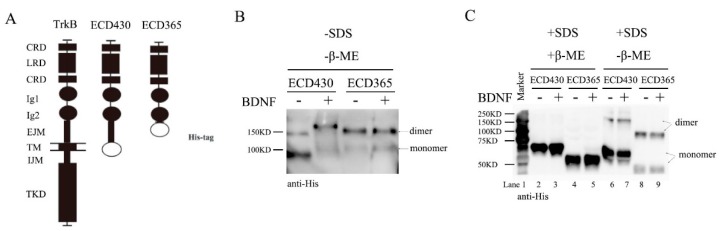
Removal of TrkB EJM facilitates dimer formation without BDNF. Recombinant TrkBECD365 or TrkBECD430 proteins (0.33 μM) were incubated with BDNF (0.33 μM) at 1:1 ratio in vitro overnight at 4 °C and Western blot was run in the presence or absence of denaturing or reducing agents. Approximately 50 ng of protein samples were loaded in each lane in either native or denaturing gels. These experiments were repeated at least three times and representative blots are shown. (**A**) Constructs of TrkB, ECD430 and ECD365. ECD430 stands for the proteins with amino acid sequence from 30 to 430 while the ECD365 means from 30 to 365 (the open ellipses indicate the poly-histidine tags). (**B**). Differential effects of BDNF on ECD dimer formation, as shown by representative native-PAGE with neither SDS nor β-ME. Note that in native conditions, TrkBECD365 formed dimer with or without BDNF, whereas for TrkBECD430, BDNF treatment facilitated the conversion from monomer to dimer. (**C**). Disruption of disulfide bond eliminate dimer formation. In denaturing gels without β-ME, substantial amount of TrkBECD365 still could form dimer, whereas TrkBECD430 was predominantly monomer unless treated with BDNF. Application of β-ME disrupted all dimer formation.

To investigate whether ECD of TrkB alone could form preformed dimers, we used Baculovirus to expressed two TrkB ECD constructs with six histidine tag in SF9 cells: TrkBECD430 with EJM and TrkBECD365 without EJM. A diagram of full-length TrkB, TrkB ECD430 and ECD365 constructs is shown in Figure 2A. The recombinant proteins were purified and incubated with BDNF (1.0 nM) overnight at 4 °C in vitro, then subjected to native gel electrophoresis. Now the bands around 90 kDa and 150 kDa correspond the TrkB-ECD monomer and dimer, respectively (Figure 2B, Supplementary Figure S2). In the native conditions with neither SDS nor β-ME, ECD430 existed mostly as a monomer. Application of BDNF converted monomer to dimer (Figure 2B left, monomer: dimer ~1:9, Supplementary Figure S2 left two lanes). This was very different from ECD365, which existed as a dimer with or without BDNF (Figure 2B right, Supplementary Figure S2 right two lanes). These experiments were performed at least three times and similar results were obtained. Thus, EJM may inhibit dimer formation and binding of BDNF to TrkBCD430 may counter the inhibitory effect of EJM.

Next, the recombinant proteins were subjected to SDS-PAGE denaturing gels. The TrkBECD430 monomer and dimer were seen as 75 kDa and 150 kDa bands, while TrkBECD365 monomer and dimer the 50kDa and 100kDa bands, respectively (Figure 2C). The molecular interactions were further investigated under two conditions: with or without reducing agent β-ME. In the presence of β-ME, both ECD430 and ECD365 became monomers, suggesting that disulfide bonds are necessary for dimer maintenance (Figure 2C, lanes 1–4). In the absence of β-ME, the majority of ECD430 became monomer, and application of BDNF only slightly increased dimer (Figure 2C lanes 6 and 7, monomer: dimer ~8:2). In contrast, ECD365 was detected more as dimer than monomer with or without BDNF (Figure 2C lanes 8 and 9, monomer: dimer ~4:6). These results again suggest an inhibitory role of EJM for dimer formation. Taken together, SDS apparently reduced the amount of ECD dimer, perhaps by suppressing electric interactions, H-H bond and Van der Waals’ force, etc. Interestingly, in the native conditions (−SDS/-β-ME), the size of ECD430 monomer appeared to be smaller than that of ECD365 (Figure 2B and Figure S2). However, in denaturing gel in the presence of SDS (+SDS/-β-ME), ECD430 monomer was larger than ECD365 (Figure 2C). These results suggest that the native TrkBECD430 monomer has a condensed 3D conformation very different from that of TrkBECD365 monomers, resulting in a smaller molecular size in native PAGE.

### 3.3. Binding of TrkB EJM by a TrkB Antibody Activates Preformed Dimers

**Figure 3:**
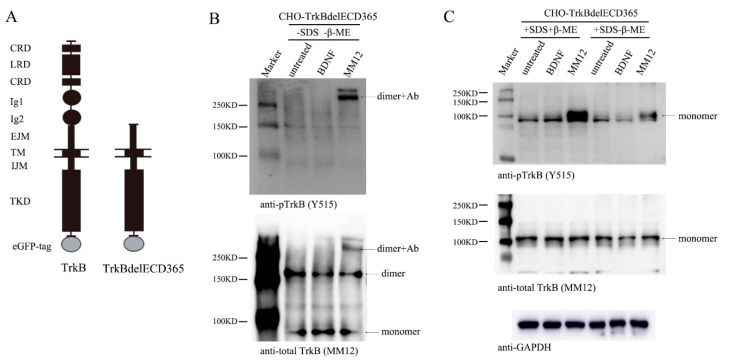
Stimulation of TrkB EJM activates preformed dimers. (**A**) Constructs of TrkB and TrkBdelECD365, both with a GFP tag. (**B**) MM12 but not BDNF induces TrkBdelECD365 tyrosine phosphorylation, only in its dimer form. Stable CHO cell line expressing TrkBdelECD365 was stimulated with BDNF or MM12 (1.0 nM, at 37 °C). Cell lysates (2 μg/lane) were subject to native-PAGE without SDS nor β-ME and Western blotting. The blots were probed anti-pTrkB (Y515) and total TrkB (MM12). (**C**) Denaturing agent SDS disrupts TrkBdelECD365 dimer formation. The experiments were done exactly the same as B except SDS, with or without β-ME, was applied to treat the cell lysates. Again, MM12 but not BDNF activates TrkBdelECD365, but only in its monomer form. The experiments in both (**B**) and (**C**) were repeated at least three times and representative blots are shown.

To examine whether the truncated TrkB lacking ECD could also exist as preformed dimers, we established a stable cell line, CHO-TrkBdelECD365-eGFP, a CHO cell line expressing human truncated TrkB, which deleted the ECD from the N-terminus to the 365th amino acid, and retained the EJM part in ECD. We named the truncated TrkB as TrkBdelECD365 (Figure 3A diagram). We used CHO cells rather than cultured neurons because the latter express endogenous TrkB that might interfere in MM12′s interaction with TrkBdelECD365. CHO-TrkBdelECD365-eGFP were treated with BDNF (1.0 nM) or MM12 (1.0 nM) for 15 min, and cell lysates were subjected to native gel electrophoresis, followed by Western blotting. The monoclonal antibody MM12 was used to detect TrkBdelECD365. Co-immunoprecipitation experiments showed that MM12 could bind full-length TrkB as well as TrkBdelECD365 (including EJM). As described above, MM12 also interacted with TrkBECD430 (including EJM) but not TrkBECD365 (excluding EJM) (data not shown). These results together indicate that MM12 could not interact with TrkB if the EJM domain is absent in TrkB, suggesting that MM12 binds specifically to TrkB-EJM.

In the native gel, the bands around 80 kDa, 160 kDa and 300 kDa correspond the monomer, dimer, and the complex of dimer plus antibody MM12, respectively (Figure 3B, bottom). About a half of TrkBdelECD365 molecules existed as dimers (Figure 3B, bottom) in the absence of ligands. Consistent with the fact that BDNF binds to the D4/D5 region of TrkB which is absent in the truncated receptor, treatment of cells with BDNF did not induce any tyrosine phosphorylation (Figure 3B, upper). Remarkably, application of the EJM-binding antibody MM12 (1nM) to the cultured CHO-TrkBdelECD365-eGFP cells induced a marked phosphorylation of the truncated receptor, TrkBdelECD365 (Figure 3B, upper,). This result suggests that the truncated TrkB could still be activated as long as the EJM domain is present. The preformed dimers in the absence of ligands were inactive (compared upper and bottom panels in Figure 3B), further suggesting that binding of MM12 to EJM could alter its conformation. Interestingly, no monomers were found to be phosphorylated, and all activated TrkBdelECD365 were dimers coupled with antibody MM12 (Figure 3B, upper). These results, which were replicated multiple times (Supplementary Figure S3A), suggest that TrkBdelECD365 also exists as a preformed dimer, which could be activated upon stimulation with the ligand to its EJM.

Finally, we examined whether the disulfide bond was necessary for the maintenance of TrkBdelECD365 dimer. The cell lysate was subjected to SDS-PAGE in the presence or absence of β-ME. TrkBdelECD365 were all shown as a 100 kDa band corresponding the monomers. No dimer was detected by SDS-PAGE, regardless of whether the reducing agent β-ME was present or not (Figure 3C). These results suggest that TrkBdelECD365 dimer was disrupted by the detergent SDS. The fact that all TrkBdelECD365 existed as monomers in SDS gel with or without β-ME further suggests that the disulfide bond was not necessary for TrkBdelECD365 dimer formation. Again, TrkBdelECD365 was not activated in cells treated with BDNF, as expected (Figure 3C, upper). In contrast, tyrosine phosphorylation could still be detected on TrkBdelECD365 monomers in cells treated with MM12 (Figure 3C, upper). Again these experiments were replicated multiple times and similar results were obtained (Supplementary Figure S3C,D).

### 3.4. TrkB ECD Does not Inhibit TrkB Activation

**Figure 4:**
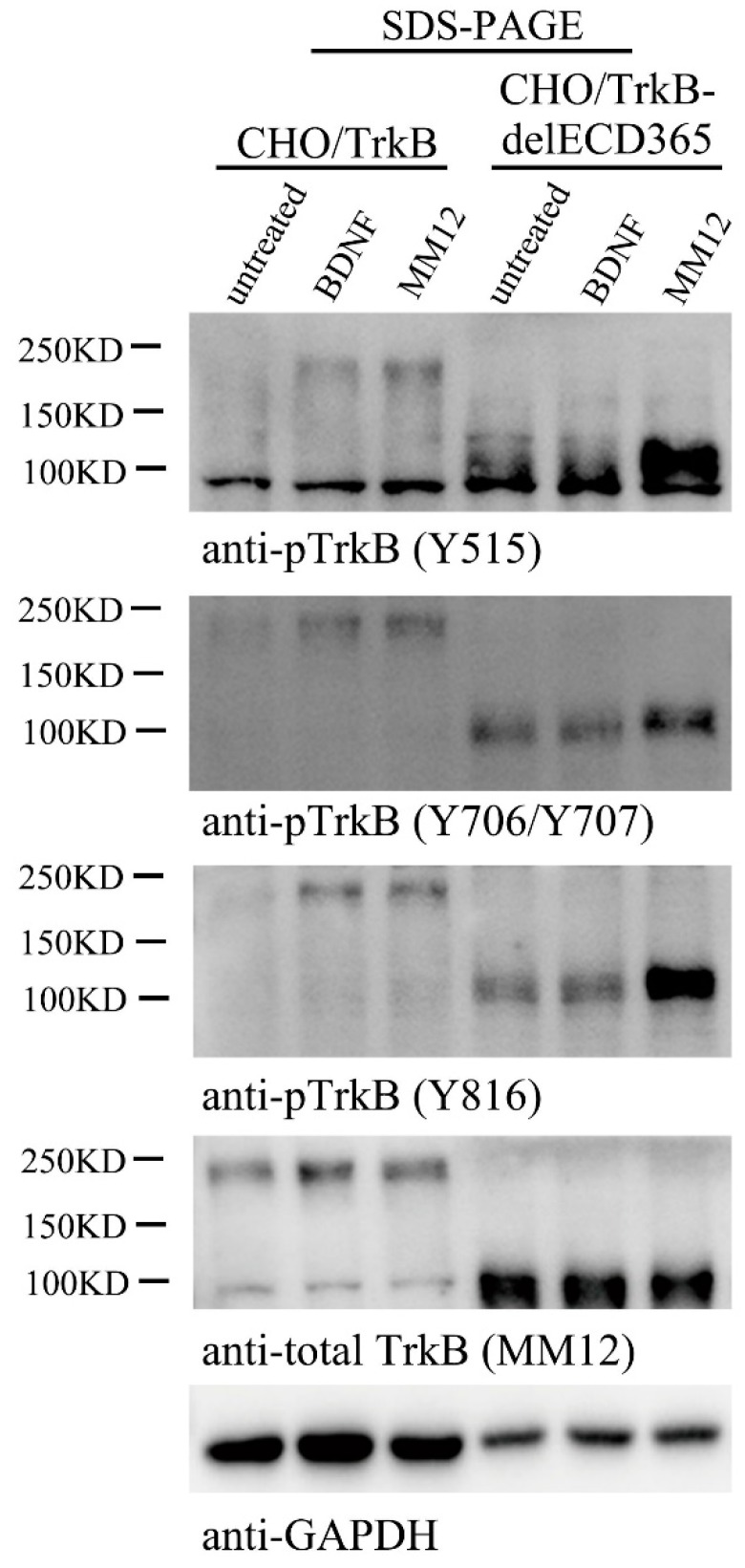
TrkBECD has no auto-inhibitory effect on TrkB activation. Stable CHO cells expressing full length TrkB (CHO/TrkB) or TrkB lacking ECD (CHO/TrkBdelECD365) were treated with vehicle, BDNF (1.0 nM), MM12 (1.0 nM) at 37 °C for 30 min, and cells were harvested, separated in SDS-PAGE, followed by Western blotting (2 μg/lane) using different anti-pTrkB antibodies. This experiment was repeated three times.

If TrkBdelECD365 itself could exist as a preformed dimer, and stimulation of the EJM domain of the truncated protein could activate its intracellular TrkB kinase, one may speculate that the entire ECD could serve as an inhibitory mechanism for TrkB activation, and binding of BDNF to ECD removes such inhibition, leading to the activation of the receptor tyrosine kinase. Indeed, for TrkA, a mutant lacking ECD was found to be constitutively active in the absence of its ligand NGF [38], suggesting that the ECD has an auto-inhibitory effect on TrkA activation. To study whether TrkB ECD also has auto-inhibitory effect on TrkB activation, we employed two stable cell lines, CHO-TrkB-eGFP which expresses the full length of human TrkB, and CHO-TrkBdelECD365-eGFP, which expresses human TrkB lacking ECD365. These cells were treated with BDNF (1.0 nM) or MM12 antibody (1.0 nM), respectively, and processed for Western blotting. Bands around 200 kDa and 100kDa correspond to full length TrkB-eGFP and TrkBdelECD365-eGFP, respectively (Figure 4). Phosphorylation of full length TrkB at sites Y515, Y706/Y707 or Y816 was induced by both BDNF and MM12 treatments (Figure 4). Although in the absence of MM12, weak phosphorylation of TrkBdelECD365 at Y706/Y707 and Y816 was detectable, treatment with MM12, but not BDNF, resulted in a marked increase in the phosphorylation of all three sites (Figure 4 and Figure S4). To confirm that these findings could be replicated in a different cellular context, we generated a PC12-TrkBdelECD365-eGFP line. Similar to that in CHO-TrkBdelECD365-eGFP, we found that MM12 but not BDNF could activate TrkB as well as its downstream signaling pathways such as pAkt in PC12-TrkBdelECD365 (Supplementary Figure S4, right 3 lanes). Thus, MM12, through binding to EJM, could activate TrkB lacking ECD365. These results, together with Figure 3B,C, suggest that unlike TrkA, TrkB-ECD does not inhibit its activation.

### 3.5. Binding of both Monomers by a Ligand Is Required for Preformed TrkB Dimer Activation

To further understand the role of EJM in TrkB activation, we took advantage of a unique feature of the monoclonal antibody: MM12 binds specifically to the EJM of human hTrkB but not rat rTrkB, and therefore activates only human but not rat TrkB. Interestingly, application of MM12 failed to activate human TrkB-eGFP with rat EJM, but could reliably activate rat TrkB-eGFP with human EJM (see below). Thus, MM12 is a specific ligand for TrkB with human EJM.

**Figure 5:**
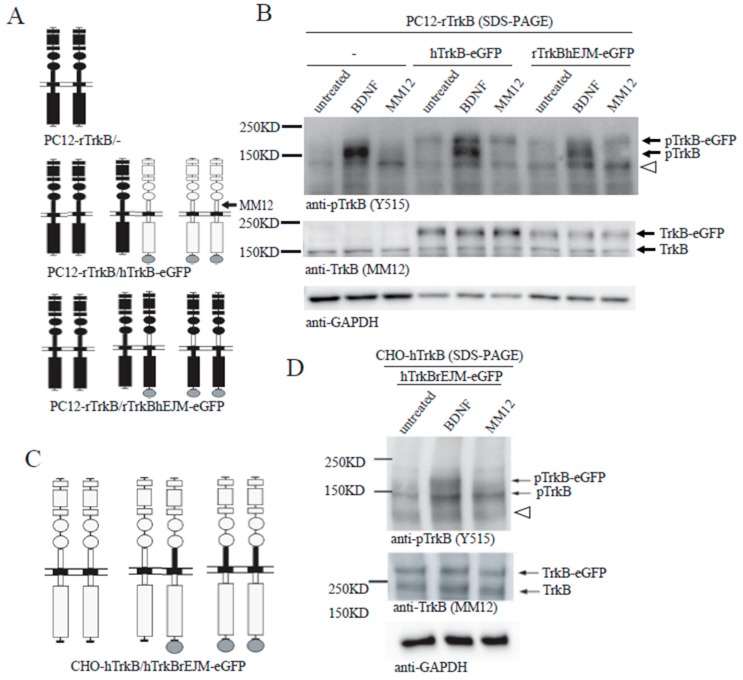
Binding of both monomers with a ligand is required for preformed TrkB dimer activation. (**A**) Schematic diagrams showing homodimer and hetero-dimer in PC12-rTrkB transfected without anything (top), with hTrkB-eGFP (middle) or rTrkBhEJM-eGFP (bottom). (**B**) MM12 selectively activates human TrkB (hTrkB) homodimer and homodimer of rat TrkB (rTrkB) with human EJM (rTrkBhEJM), but not hTrkB-rTrkB hetero-dimer or rTrkB- rTrkBhEJM hetero-dimer. PC12 cells expressing endogenous rTrkB were transfected with hTrkB-eGFP or rTrkBhEJM-eGFP, stimulated with vehicle, BDNF, or MM12 (1.0 nM) for 15 min before harvesting. Cell lysates (2 μg) were subject to SDS-PAGE and processed for Western blotting. The blots were probed anti-TrkB (Y515) for pTrkB and MM12 for total TrkB. Open triangle points to a non-specific band. (**C**) Schematic diagrams showing hTrkB homodimer (left), hTrkB- rTrkBhEJM-eGFP hetero-dimer (center) and rTrkBhEJM-eGFP homodimer. (**D**) MM12 selectively activates hTrkB, but not a TrkB dimer containing either one or two copies of rEJM. CHO cells were transfected with both hTrkB and hTrkBrEJM-eGFP, stimulated with BDNF or MM12, and then processed for SDS-PAGE and Western blotting the same way as C. Note that MM12 only activate hTrkB homodimer (lower molecular weight band), but not the hTrkBrEJM-eGFP containing hetero- or homo- dimers (higher molecular weight band). Open triangle points to a non-specific band. Each experiment was repeated twice.

We designed a domain-swap experiment using PC12 line, which does not express endogenous TrkB that could potentially interfere with the engineered rat or human TrkB. A PC12-rTrkB line expressing endogenous rat TrkB (short) was stably transfected with hTrkB-eGFP (long). If all TrkB exists as a preformed dimer, this line should express three types of TrkB dimers: rTrkB/rTrkB homo-dimer, hTrkB-eGFP/hTrkB-eGFP homo-dimer, and rTrkB/hTrkB-eGFP hetero-dimer, as shown in Figure 5A (middle). The human and rat TrkB molecules could be distinguished by Western blot according to the molecular weight difference of eGFP, 27 kDa. In a control experiment using PC12-rTrkB line, application of BDNF, but not MM12, induced rTrkB phosphorylation (Figure 5B, left 3 lanes). In PC12-rTrkB line transfected with hTrkB-eGFP, treatment with BDNF resulted in phosphorylation of both rTrkB and hTrkB. In contrast, treatment with MM12 elicited phosphorylation of only hTrkB but not rTrkB (Figure 5B, middle 3 lanes). This result suggests that MM12 could not induce tyrosine phosphorylation of either rTrkB homo-dimer or rTrkB/hTrkB-eGFP hetero-dimer. Should some inter-molecular phosphorylation occur, at least some tyrosine phosphorylation of the rTrkB should be detected. The inability of MM12 to activate the preformed dimer of rTrkB/hTrkB-eGFP hetero-dimer also implies that binding of MM12 to one copy (hTrkB-eGFP) of TrkB in the hetero-dimer was not sufficient to trigger rTrkB activation.

Given that MM12 selectively binds the EJM domain of hTrkB, we further asked whether interaction of MM12 with EJM on one or both copies of the TrkB dimer is necessary for the receptor activation. A domain-swap experiment was designed: PC12-rTrkB line stably transfected with rTrkBhEJM, in which the rat EJM was replaced by human EJM. This line should express three types of TrkB dimers: rTrkB/rTrkB homo-dimer, rTrkBhEJM-eGFP/rTrkBhEJM-eGFP homo-dimer, and rTrkB/rTrkBhEJM-eGFP hetero-dimer, as shown in Figure 5A (bottom). BDNF, which binds to the D4/D5 region of TrkB, was able to induce tyrosine phosphorylation of both rTrkB and rTrkBhEJM-eGFP (Figure 5B, right and Supplementary Figure S5). MM12, however, was able to activate only rTrkBhEJM-eGFP, but not rTrkB (Figure 5B, right). This result again suggests that MM12 could only bind and activate the rTrkBhEJM-eGFP homo-dimer, but not the rTrkBhEJM-eGFP-rTrkB hetero-dimer.

To validate this finding in a different system, similar domain-swap experiments were performed using CHO cells transiently co-transfected with hTrkB and hTrkBrEJM-eGFP (Figure 5C). This line should express hTrkB/hTrkB homo-dimer, hTrkB-/hTrkBrEJM-eGFP hetero-dimer, and hTrkBrEJM-eGFP/hTrkBrEJM-eGFP homo-dimer, Again, application of BDNF activated both hTrkB and hTrkBrEJM-eGFP. In contrast, application of MM12, which binds human but not rat EMJ, induced tyrosine phosphorylation of only hTrkB (lower molecular weight band) but not hTrkBrEJM-eGFP (higher molecular weight band) (Figure 5D). Taken together, these results further support the notion that MM12 needs to bind to EJM domains on both subunits of TrkB in order to activate it via preformed dimers. Thus, binding of both monomers by a ligand is required for preformed TrkB dimer activation.

## 4. Discussion

**Figure 6:**
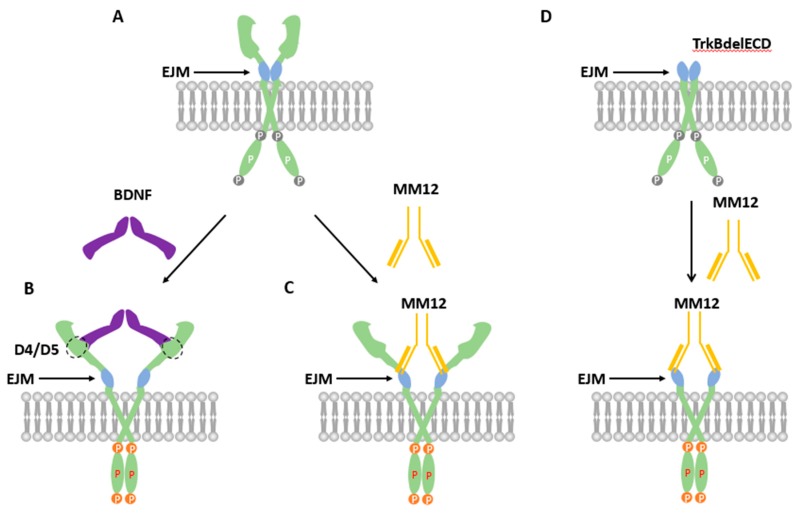
Hypothetic models depicting how BDNF and the EJM-binding antibody MM12 could activate the preformed TrkB dimer. (**A**) Inactive preformed dimer of TrkB. Note that the two intracellular kinase domains (IKCs) are separated from each other, making it difficult for cross-phosphorylation. (**B**) TrkB activated by BDNF. BDNF binds the D4/D5 region on TrkB, pushing the two ECDs apart while bringing the two IKCs together. This results in inter- molecular phosphorylation and TrkB activation. (**C**) TrkB activated by MM12. The agonistic antibody MM12, through interaction with the TrkB-EJM, also activates TrkB by conformational changes that brings the intracellular kinase domains (IKDs) closer. (**D**) TrkBdelECD365 activated by MM12. Without ECD, MM12 could still induce the similar conformation changes that brings IKDs together, leading to its activation.

It is generally believed that RTKs are activated by ligands through a ligand-induced dimerization [6,39]. However, this model has been challenged. For example, a number of studies have demonstrated that the EGF receptor ErbB exists as inactive dimers on cell surface before ligand binding [7,8,9,10,11,12,13,14,15,16]. These results support a different view: the unliganded RTKs could form an inactive dimer, which could be activated through an “rotation-activation” mechanism [6,39]. Our previous work has shown inactive and preformed dimers in living cells in the neurotrophic tyrosine kinase receptors, TrkA and TrkB, in heterologous expression systems [34,35]. Whether application of BDNF can fully activate the kinase activity of the preformed dimer was not fully established. Further, these studies have only examined the role of intracellular domain of TrkB in dimer formation. In the present study, we showed that both TrkB-ECD and CHO-TrkBdelECD can form preformed dimer, and that EJM domain appears to have an inhibitory effect on dimer formation. More importantly, application of the TrkB ligand BDNF or TrkB agonistic antibody (MM12) activates the pre-existing dimer by phosphorylating all the critical tyrosine residues as demonstrated by Western blots using specific anti-tyrosine antibodies. These results together support the model that the preformed dimer could be activated by its ligand BDNF dimer through attenuation of the inhibitory effect of EJM (Figure 6). In the resting state, EJM sets a conformation in which the two intracellular kinase domains (IKDs) are separated (Figure 6A). Binding of BDNF to the D4/D5 region triggers a conformational change that counters the inhibitory effects of EJM. This brings the two IKDs together, resulting in inter-molecular phosphorylation and TrkB activation (Figure 6B). Binding of MM12 to EJM domain of TrkB results in similar conformation change and TrkB activation (Figure 6C). When TrkB ECD is removed, binding of MM12 to EJM could still bring the two IDKs together and elicit TrkB RTK activation (Figure 6D). These results provide new insights into the preformed dimer model for TrkB. Whether this model could be applied to other RTK requires further investigations.

While our results demonstrated that in cultured neurons, a substantial amount of endogenous TrkB exists as an inactive preformed dimer, a recent study has provided data that suggest a different model. Using a combined single-molecule, super-resolution imaging, and FRET and TIRF technologies, Zahavi et al. reported that in HEK cells and in embryonic spinal cord neurons, transfected TrkB could be activated mildly as a monomer on the plasma membrane upon binding to BDNF, leading to the activation of Erk1/2 signaling [40]. After internalization, it forms a dimer within BDNF-containing endosomes, leading to much stronger activation, particularly that of the PI3K-Akt pathway. This is a very provocative model that seems to be different from many of previously published results as well as our current data, which were largely obtained through biochemistry experiments. Taking advantages of the special feature that MM12 antibody binds only to EJM of human (h) but not rat (r) TrkB, we performed “swap” experiments in which rEJM was replaced with hEJM and vice versa. We found that MM12 could only activate TrkB when both monomers contain hEJM, suggesting that binding of two EJM on dimer is required for TrkB activation (Figure 5). MM12 but not BDNF induced TrkBdelECD365 tyrosine phosphorylation, only in its dimer form. Using a stable CHO cell line expressing TrkBdelECD365, we found MM12 but not BDNF induced TrkBdelECD365 tyrosine phosphorylation, only in its dimer form, detecting pTrkB with native PAGE without SDS nor β-ME, and no phosphorylated TrkB delECD365 was detected (Figure 3). More importantly, using native, non-reducing gels and multiple TrkB antibodies, we showed that endogenous TrkB was expressed largely as preformed dimers in primary hippocampal neurons (Figure 1). These results are in contrast to the data by Zahavi et al. [40]. However, we found that BDNF and MM12 could activate TrkB and TrkBdelECD365, leading to the PI3-Akt pathway (Supplementary Figure S4). Thus, it remains possible that the monomer activation Zahavi et al. described is a special case in which TrkB signaling remains on plasma membranes without endocytosis. It is also unclear whether the differences in the methods used (imaging versus biochemistry), endogenous versus over-expression, or the antibodies used, have contributed to the discrepancies. Future experiments combining different technologies will be necessary to resolve these discrepancies.

BDNF itself exists as a dimer endogenously, which naturally would be more likely to bind TrkB dimer rather than monomer. More recently, Ahmed and Hristova studied dimer formation of all Trks using FRET-based techniques [41]. They showed that unliganded Trks could exist as preformed dimers. Moreover, ligand binding increases Trk dimer stability and induces conformational changes that are propagate to Trk intracellular domains. It remains to be determined whether endogenous TrkB forms an inactive dimer in living neurons without over-expression. The present study has demonstrated by native-PAGE that endogenous unliganded TrkB forms inactive dimers in cultured neurons. These results provide further support for the “preformed dimer” model, at least for TrkB (Figure 6).

Previous studies have shown that when immunoglobulin-like domain 1 (Ig1, D4), Ig2 (D5) or both Ig1 and Ig2 of TrkA were deleted, significant spontaneous dimerization of TrkA occurred in the absence of NGF. In contrast, the dimerization of the wild-type TrkA occurred only in the presence of NGF, indicating that both Ig domains had inhibitory effect on the dimer formation [39]. Removal of the two Ig domains rendered TrkB constitutively active, suggesting that the two Ig domains on TrkB may have a similar effect [42]. The present study has provided further insights. First, in native PAGE gels (Figure 2B), all TrkBECD365 (TrkB ECD lacking EJM) existed as a preformed dimer while only a small fraction of TrkBECD430 (TrkB ECD containing EJM) was detected as dimer, suggesting the inhibitory effect of EJM on TrkB dimerization. However, a 280 kDa band of dimer of full-length TrkB was consistently detected by multiple TrkB antibodies in lysates from cultured primary neurons resolved in native gels (e.g., 80E3 and MM12 in Figure 1B). This implies that TrkB ECD without EJM may promote its dimerization. Second, in native PAGE, almost all TrkBECD365 molecules were found as dimers, suggesting that Ig domains could not inhibit TrkBECD365 dimerization (Figure 2B, right). On the other hand, TrkBECD430 existed primarily as a monomer, and application of BDNF converted it to dimer (Figure 2B, left). Thus, the EJM inhibited the dimerization of TrkBECD430. Taken together, it appears that the inhibitory effect of EJM may be stronger than that of Ig domains. Third, in both denaturing and non-denaturing gels in the absence of the reducing agent β-ME, only a small fraction of ECD430 was detected as a dimer (Figure 2B,C). This result suggests that the dimerization of the entire TrkB ECD in the absence of ligand results from a balance between inhibitory and promoting effects of different domains, and the entire TrkB ECD may not be easy to dimerize. Consistently, using luciferase complementation assay, it was shown that removal of intracellular domain disrupted TrkB preformed dimer formation [35]. We could further infer that, as for the full length of TrkB in the absence of ligands, the intracellular domain may contribute to its dimerization more than the extracellular domain.

What are the molecular forces that mediate the interaction between two preformed dimers? Disulfide bonds were reported to be important for the formation of preformed dimers in another type of neurotrophic factor receptor, p75NTR [43,44]. By SDS-PAGE in the absence or presence of reducing regent β-ME, we have shown that disulfide bonds are also necessary for the preformed dimers of full length of TrkB or TrkB ECD, but not TrkBdelECD365 (Figure 1B). There are thirteen cysteines in TrkB ECD and form six disulfide linkages as Cys1-Cys7, Cys5-Cys14, Cys121-Cys145, Cys123-Cys163, Cys187-Cys235, and Cys271-Cys314, respectively. Only Cys300 is a free sulfhydryl residue [45]. More investigations are needed to determine which cysteine(s) is (are) important to dimerization. Moreover, SDS was found to disrupt part of the dimers (Figure 1B and Figure 2C). In the absence of β-ME, application of SDS decreased the TrkBECD430 dimer/monomer ratio (Compare Figure 2B,C). These results together suggest that molecular forces other than disulfide bond are involved in the interaction of dimers.

Why could the TrkB preformed dimer be activated? It is surmised that in the absence of its ligand, some structures such as the Ig domains and EJM in the preformed dimer would impede the receptor being spontaneously activated. As discussed earlier, TrkB lacking Ig domains is constitutively activated, triggering the downstream Erk1/2signaling [42]. We now show that deletion of the EJM facilitated the dimer formation (Figure 2B,C). Interestingly, binding of the EJM with an EJM-specific antibody (MM12) also enhanced dimer formation and even resulted in its activation (Figure 3B,C and Figure 4). It is tempting to speculate that the binding of the EJM by MM12, similar to EJM deletion, could remove the inhibitory effects of EJM and induce the conformation changes, leading to TrkB activation (Figure 6).

Altogether, this study has shown, for the first time, that inactive and preformed dimers of endogenous TrkB exist in cultured neurons. We demonstrated that both extracellular domain and intracellular domain contribute to dimerization, while the contribution of ECD is weakened by the inhibitory effect of EJM. Further studies are necessary to understand how the preformed dimer of TrkB maintains its stability and how the ligand induces the conformation change of preformed dimers.

## Supplementary Materials

The following are available online at https://www.mdpi.com/2073-4409/8/8/932/s1.

## Abbreviations

BDNF: Brain-derived neurotrophic factor; ECD: Extracellular domain; DPBS: Dulbecco’s phosphate-buffered saline; eGFP: enhanced green fluorescent protein; ICD: intracellular domain; EJM: Extracellular juxtamembrane motif; mAb: monoclonal antibody; NT: neurotrophin: Trk: tropomyosin-related kinase; PAGE: polyacrylamide gel electrophoresis; SDS: sodium dodecyl sulfate; CRD: cysteine-rich domains; LRD: leucine-rich domain; Ig1: immunoglobulin-like domain 1; Ig2: immunoglobulin-like domain 2; GAPDH: Glyceraldehyde 3-phosphate dehydrogenase; BSA: bovine serum albumin; IKDs: intracellular kinase domains; RTKs: receptor tyrosine kinases; β-ME: β-mercaptoethanol.
