# Supplementary material for: Extracellular Juxtamembrane Motif Critical for TrkB Preformed Dimer and Activation
Source: Cells. 2019 Aug 19;8(8):932. doi: 10.3390/cells8080932 (PMC6721692; doi:10.3390/cells8080932)
Supplement: Supplementary file 1 [file cells-08-00932-s001.pdf]

cells

Supplementary Data

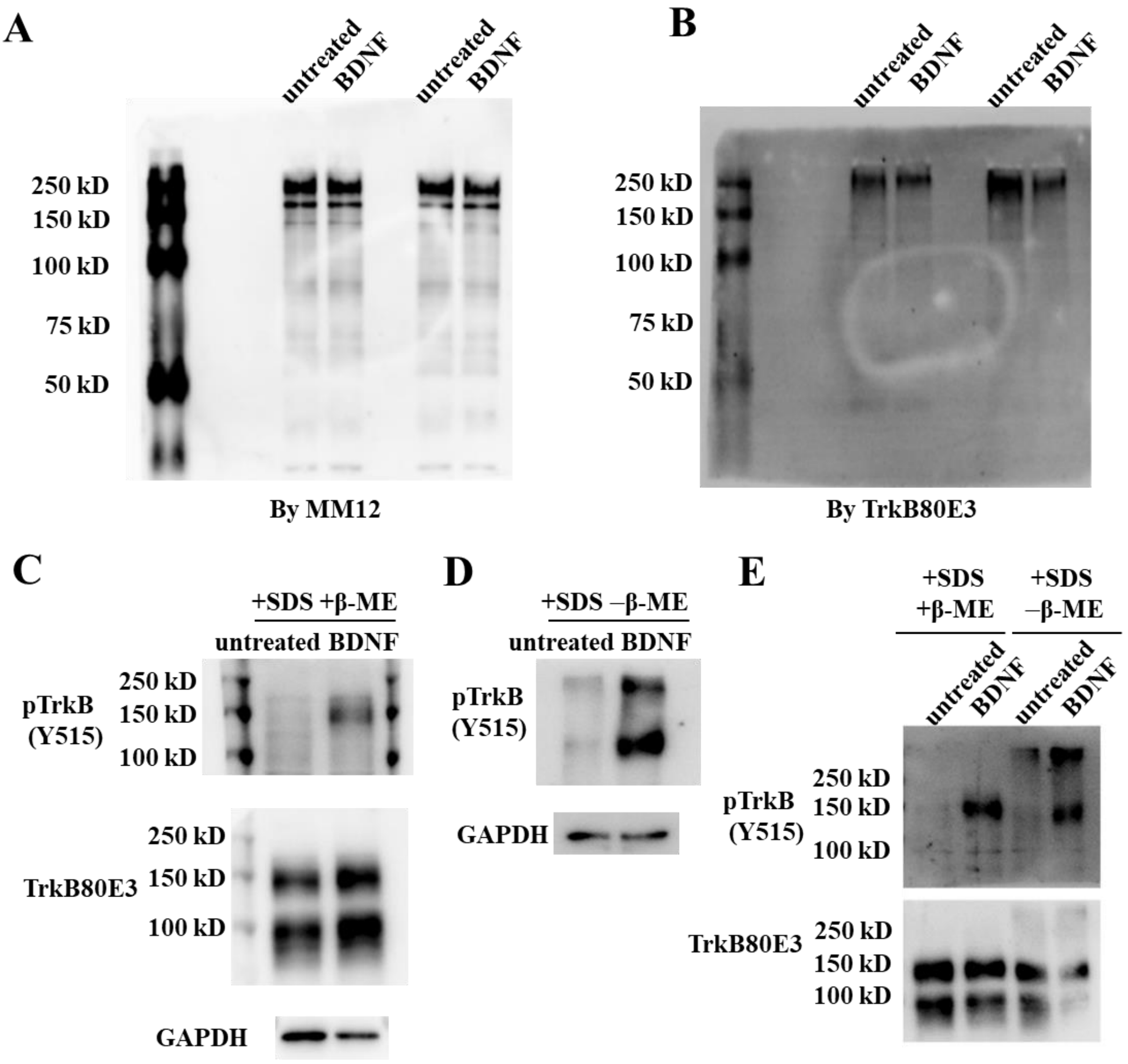

Supplementary Fig. 1. TrkB preformed dimer and its activation by BDNF. Rat hippocampal neurons (10 days in culture) were treated with or without BDNF (1.0 nM) for 15 min. and processed for Western blotting. (A) and (B) Cultures were harvested and proteins were separated in native gels (in the absence of SDS, without β-ME), and probed anti-TrkB antibodies MM12 (A) or 80E3 (B). The same experiment as Fig. 1A, middle, right 2 lanes, Note that TrkB existed primarily as a preformed dimer before BDNF stimulation. (C) and (D) The experiments were carried out in SDS with (C) or without (D) β-ME, and the Western blot was probed with the anti-pTrkB (Y515) or the TrkB80E3 antibody. The results are the same as Fig. 1B, middle, showing activated TrkB monomer and dimer. (E) The experiment was carried out in SDS with (left 2 lanes) or without (right 2 lanes) β-ME, and the Western blot was probed with anti-pTrkB (Y515) (upper) as well as TrkB80E3 (lower).The experiment was essentially the same as Fig. 1B, middle and upper, showing activated TrkB monomer and dimer.

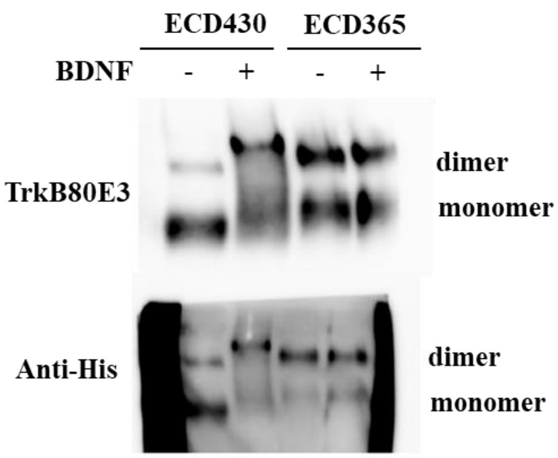

Supplementary Fig. 2. BDNF increases dimer formation for ECD430 but not for ECD365. The same experiment as Fig. 2B, showing the effect of BDNF TrkB dimer formation. The experiment was carried out in the absence of SDS and β-ME, and the Western blot was probed with TrkB80E3 and anti-His antibodies.

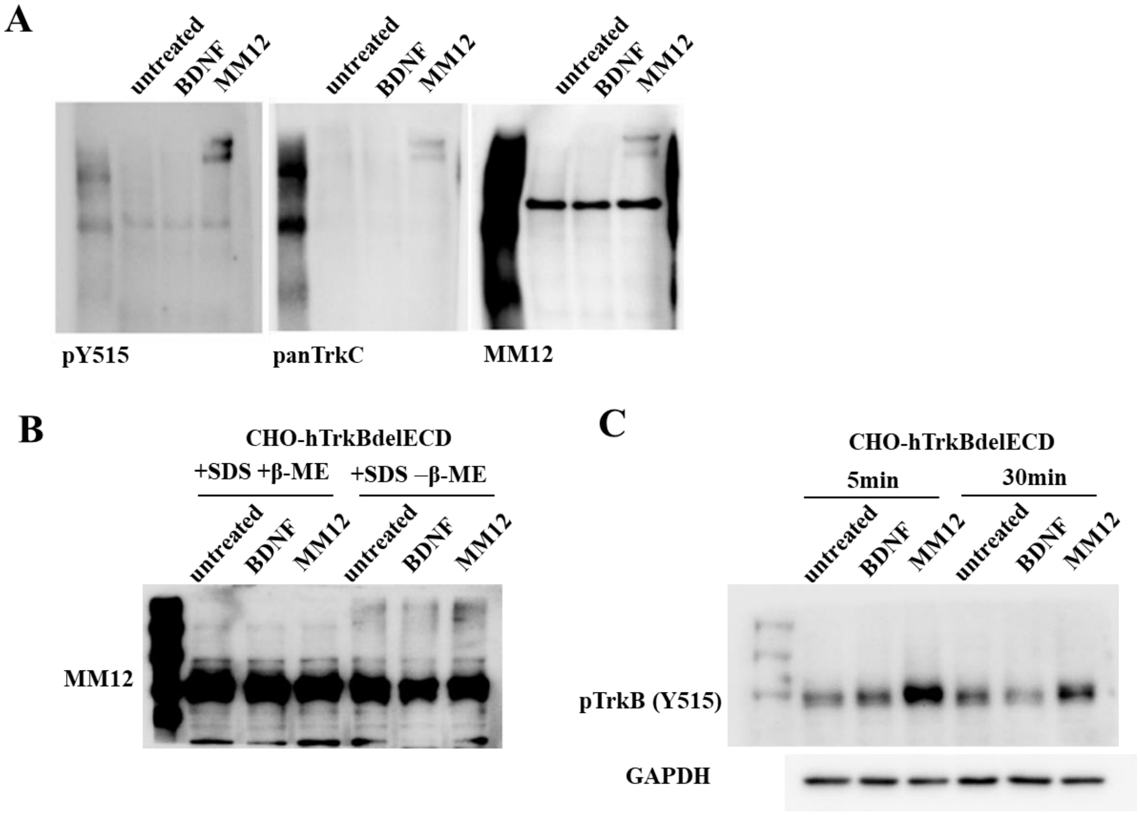

Supplementary Fig. 3. Activation of preformed TrkB dimer by stimulating TrkB EJM. (A): Lysates (2 µg/lane) derived from TrkBdelECD365-expressing cells were subject to native gel (-SDS -β-ME), and the Western blots were probed with anti- pTrkB(Y515) (left), a pan Trk (middle), and MM12（right） antibodies. The results are essentially the same as Fig. 3B. (B) and (C): The same as A, except the experiments were carried out in the presence of SDS, with or without β-ME as indicated, and the Western blot was probed with MM12 antibody (B) and anti-pTrkB(Y515) antibody (C). The results are essentially the same as Fig. 3C, carried out in the presence of SDS and β-ME, showing MM12, but not BDNF, could activate TrkBdelECD365.

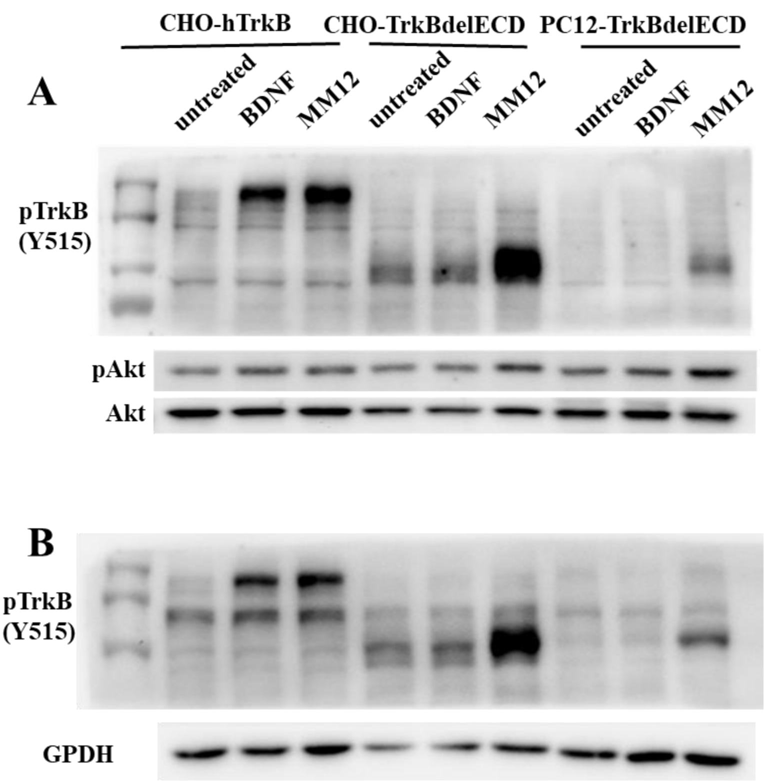

Supplementary Fig. 4. Deletion of TrkB-ECD does not affect TrkB activation. The experiments were carried essentially the same way as that in Fig. 4. Stable CHO cells expressing TrkB (CHO-hTrkB) or TrkB lacking ECD (CHO-hTrkBdelECD365), as well as PC12 cells stably expressing TrkBdelECD365, were used. Cells were harvested after treatment with BDNF or MM12 for 30 min, followed by Western blots. (A) The blot was probed pTrkB, pAkt, Akt antibodies. Note that for both CHO and PC12 lines expressing TrkB lacking ECD, treatment with the TrkB agonist antibody MM12 but not BDNF led to the activation of this truncated TrkB and its downstream pAkt. (B) An independent experiment identical to that of (A). The results in this figure are essentially the same as Fig. 4.

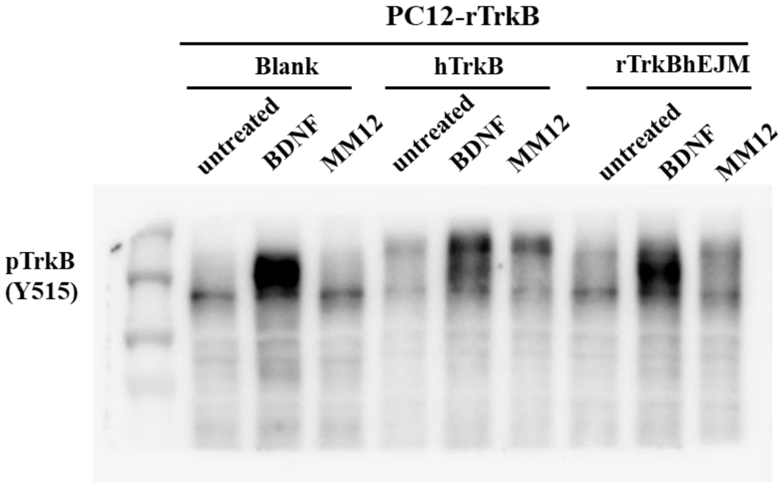

Supplementary Fig. 5. MM12 selectively activates human TrkB (hTrkB) homodimer and homodimer of rat TrkB (rTrkB) with human EJM (rTrkBhEJM), but not hTrkB-rTrkB

hetero-dimer or rTrkB- rTrkBhEJM hetero-dimer. The experiment was exactly the same as that in Fig. 5B (top). The blot was probed with pTrkB (Y515).
